# Health Tract, No. 193

**Published:** 1864-02

**Authors:** 


### BREAD.

CONSIDERING that not one hired cook in a thousand makes good bread, it is more healthful to use baker's bread, and is also more economical for small families. Baker's bread is always good, fresh, and light, hence there is no waste. To prevent waste where home-made bread is used, it being so often heavy, hard, burnt, or sour, it is made into toast, or bread pudding, whch requires so much sweetening and butter and spices that the "saving" is all lost. An intelligent and observant writer states in that excellent monthly, the *American Agriculturist*, that his family of five persons paid out in one year for

| | | Another year. | |
|---|---|---|---|
| Meats, | $95 68 | Meats, | $84 76 |
| Flour, 5 barrels, | 46 25 | Bread, 451 loaves, 5c., | 61 30 |
| Butter, 22½c., | 65 81 | Butter, | 56 81 |
| Total, | $207 74 | Total, | $202 87 |
| Flour and butter, | $112 06 | Bread and butter, | $118 11 |

The bread cost more, but the butter less, and less meat was eaten, to say nothing of the fuel, time, milk, salt, rising, etc. saved by using baker's bread; then, there is the comfort of having good bread always on the table, and the absence of that annoyance which an intelligent mind always experiences when compelled to eat unwholesome food. The amount of injury done to the tender stomachs of young children, invalids, and sedentary persons, by eating bad bread day after day, from one year's end to another, must be enormous. A cook who can not make good bread of every description, ought not to be allowed house-room for an hour; and that mother is criminally negligent, whatever may be her position, who does not teach her daughter to know what good bread is; and also how to make it. Alum is used to give whiteness, softness, and capacity for retaining moisture. Lime could be employed with equal effect, having the advantage of correcting any sourness in the bread or stomach; besides affording an important ingredient for making the bones strong. Every housekeeper ought to know how to make two or three kinds of bread. The best yeast in the world is made of hops and cold water, nothing else. If lime-water is used, it should be water saturated with lime, that is, holding as much lime as it can; if it has for a moment more, it goes to the bottom, as sugar in a tea-cup, when the tea can be made no sweeter. Use nineteen pounds of flour and five pounds of saturated lime-water made thus: Put stones of quick lime in water, stir until slack, let it settle and then pour off. Soda (an alkali made of sea-salt) and saleratus (an alkali made of wood ashes) are used for the self-same purpose, to neutralize any sourness in the bread; one is in no respect better than the other; but as cooking soda is the cheapest, it is an economy to prefer it.

JOHNNY CAKE.—To two quarts of Indian (corn meal) add a tea-spoonful of salt and as much cold water as will make a soft dough; bake one hour, eat hot, with milk. Stir cold water into unsifted wheat meal until a not very soft batter is made, put into small patty-pans, bake in a hot oven half an hour or more.

RAISED BISCUIT.—A pint of light dough, a fresh egg and its bulk of fresh butter. Knead most thoroughly for ten minutes; roll out and let them rise on a shallow pan, in a moderately warm place for half an hour; bake for twelve or fifteen minutes in a hot oven; to be eaten while fresh. The two most important requisites for making good bread are a most patient and thorough kneading and a hot oven, kept steadily hot, until the baking is completed.

PATENT FLOUR is made of the following drugs: With six pounds of wheat flour mix five tea-spoonfuls of cooking-soda, then seven of cream of tartar and six of common salt. Shorten or not with a quarter of a pound of butter.

